# Gaze Patterns in Auditory-Visual Perception of Emotion by Children with Hearing Aids and Hearing Children

**DOI:** 10.3389/fpsyg.2017.02281

**Published:** 2017-12-22

**Authors:** Yifang Wang, Wei Zhou, Yanhong Cheng, Xiaoying Bian

**Affiliations:** ^1^School of Psychology, Capital Normal University, Beijing, China; ^2^Wuhan Children’s Library, Wuhan, China

**Keywords:** hearing-impaired, emotion perception, eye-movement, auditory-visual perception, oral statement

## Abstract

This study investigated eye-movement patterns during emotion perception for children with hearing aids and hearing children. Seventy-eight participants aged from 3 to 7 were asked to watch videos with a facial expression followed by an oral statement, and these two cues were either congruent or incongruent in emotional valence. Results showed that while hearing children paid more attention to the upper part of the face, children with hearing aids paid more attention to the lower part of the face after the oral statement was presented, especially for the neutral facial expression/neutral oral statement condition. These results suggest that children with hearing aids have an altered eye contact pattern with others and a difficulty in matching visual and voice cues in emotion perception. The negative cause and effect of these gaze patterns should be avoided in earlier rehabilitation for hearing-impaired children with assistive devices.

## Introduction

Decoding people’s emotional expression is essential for a human’s successful social interaction (Lopes et al., 2004; Smith et al., 2005). Emotion can be judged by examining the face, voice, and body (Rosenberg and Ekman, 1994; Scherer, 2003; Banziger et al., 2009). Human faces, especially the eyes, are the primary and most powerful mediation for the perception and communication of emotion (O’Donnell and Bruce, 2001; Schyns et al., 2002; Vinette et al., 2004; Adams and Nelson, 2016). However, one cannot accurately interpret other’s emotion only through facial cues because there may be conflicting information in voice cues. An example of such a case is sarcasm (Zupan, 2013). By matching both auditory and visual information, people can better understand others’ emotional and mental states. Consequently, the development of social competence largely depends on learning to integrate and interpret facial and voice cues of emotion (Mayer et al., 2004).

Due to early sensory impairment, young children with severe and profound hearing loss lack auditory information in emotional communication and have difficulties in adult-child interactions. This implies that hearing-impaired children have a risk of deficits in understanding emotional expressions (Rieffe and Terwogt, 2000). Currently, an increasing number (i.e., 25% average annual growth rate in China since 1995, Liang and Brendan, 2013) of hearing-impaired children wear assistive devices [e.g., digital hearing aids (HAs) or cochlear implants (CIs)] to improve hearing and speech. As a result, there is a need to explore the effects of using assistive devices on children’s emotional development.

Previous research on emotion perception by children with assistive devices has mainly used emotion matching or labeling tasks to measure the response accuracy in recognizing facial expressions of different emotions. While some studies on children have found that the hearing group did not show any advantage in facial expression recognition over the severely and profoundly hearing-impaired group with CIs or HAs (Hosie et al., 1998), a growing body of research has shown that hearing children perform significantly better in facial expression recognition than those with CIs or HAs (Most et al., 1993; Gray et al., 2001; Dyck et al., 2004), suggesting that earlier intervention for severely and profoundly hearing-impaired children should include not only language aspects but also emotional treatment (Wang et al., 2011; Wiefferink et al., 2013).

Facial muscle movements provide a perceptual basis for identifying different emotional expressions. For example, the facial expression of happiness can be characterized as a flexing of the mouth muscles and a restriction of the eyes (Ekman and Friesen, 1978). To measure how a human deploys attention during facial expression decoding, researchers have utilized eye-movement techniques to investigate gaze tendencies in different parts of the face (e.g., Aviezer et al., 2008; Schurgin et al., 2014). Eye-movement studies on individuals with autism spectrum disorder and social anxiety disorder have demonstrated that participants with deficits look less at others’ faces, particularly the eye regions, compared to hearing controls (Klin et al., 2002; Horley et al., 2003, 2004; Riby and Hancock, 2008; Nakano et al., 2010; Weeks et al., 2013; Falck-Ytter et al., 2015). These results suggest that deficits in emotional and social communication are associated with gaze avoidance (i.e., avoiding looking at others’ eyes). For people with hearing loss, previous studies have indicated that they have different eye-movement patterns in face-to-face communication. While some research has indicated that deaf people rely more on visual cues in the eye regions relative to hearing people (Luciano, 2001), other studies have found that severely and profoundly hearing-impaired people pay more attention to the lower part of the face (e.g., Muir and Richardson, 2005; Letourneau and Mitchell, 2011).

Deficits in emotion perception among severely and profoundly hearing-impaired people are also associated with their deprivation of auditory-linguistic experience (Gray et al., 2001; Geers et al., 2013). On one hand, auditory experience in the first period of life is critical for the development of speech perception and production (Kral, 2007), which are fundamental for voice emotion recognition. Some studies have reported that voice emotion recognition in both congenitally and progressively hearing-impaired individuals with assistive devices is rather poor, as compared to their hearing peers (Hopyan-Misakyan et al., 2009; Volkova et al., 2013; Wang et al., 2013). On the other hand, auditory deprivation may also influence the remaining visual and somatosensory modalities (Gori et al., 2017), which are fundamental for face expression perception.

Emotion perception requires the matching of both visual and auditory information (Mayer et al., 2004). However, studies of emotion perception focused on hearing-impaired children’s performance in visual face recognition or voice emotion recognition separately. Without considering emotional aspects, previous studies have found that deaf individuals with restored hearing produced different behavioral patterns as compared to hearing controls in audio-visual integration tasks (e.g., Doucet et al., 2006; Rouger et al., 2008; Bergeson et al., 2010; Gori et al., 2017), suggesting a cross-modal reorganization at the cortical level for them (Doucet et al., 2006). Likewise, it is necessary to investigate hearing-impaired people’s behaviors when both visual and auditory cues of emotion are available in a typical social context (Ziv et al., 2013). Most et al. (1993) created auditory only, visual only, and auditory-visual modalities of emotional expressions (e.g., anger, disgust, surprise, and sadness) to study emotion perception for hearing-impaired (with HAs) and hearing adolescents. They found that the recognition accuracy in the auditory-visual mode was higher than that in the auditory or the visual modes alone for the hearing participants, but there was no difference between the auditory-visual mode and the visual mode alone for the hearing-impaired participants. This result was replicated by Most and Aviner (2009) and suggested that adolescents with profound and congenital hearing loss were mainly relying on visual information to interpret emotional expressions. However, when Most and Michaelis (2011) tested preschoolers (4.0–6.6 years) as participants, they found that the accuracy of emotion perception in auditory-visual conditions was significantly higher than in auditory or visual modes alone for both hearing children and children with hearing loss ranging from moderate to profound, indicating that hearing-impaired young children utilized both visual and auditory information for emotion perception.

As indicated above, different studies have used different age-samples (e.g., preschoolers and adolescents), different modalities of stimuli (e.g., auditory only, visual only, and auditory-visual), and different measurements (e.g., response accuracy and eye-movement patterns) to investigate hearing-impaired children’s deficits in emotion perception. However, there is still a lack of research to examine the eye-movement patterns of hearing-impaired preschoolers in a relatively ecological context for emotion perception. To construct a more valid context for emotion perception, Weeks et al. (2013) adopted a dynamic *social evaluation task*, wherein participants were asked to watch a video with a static facial expression followed by a talking face with an oral statement to assess the gaze behaviors of social anxiety disorder. We used similar materials and a similar task in the current study. The stimuli included the positive or neutral facial expression as visual cues and the positive or neutral oral statement as voice cues, which were either congruent or incongruent in emotional valence with each other. When hearing-impaired children with HAs had deficits in emotional expression recognition, they would produce different gaze patterns (e.g., reduced eye contact) as compared to hearing children. Specifically, if different gaze patterns existed during the static face period, the deficits would be ascribed to only visual aspect. In contrast, if different gaze patterns existed during the oral statement period, the deficits would be ascribed to the matching of visual and auditory information. In addition, whether the group effect was modulated by the consistency between visual and voice cues would validate hearing-impaired children’s interactive deficits in emotion perception.

## Materials and Methods

### Participants

Thirty-nine preschool-aged hearing-impaired children (*M_age_* = 60 months, *SD* = 12; 24 boys and 15 girls) with HAs and 39 hearing children (*M_age_* = 62 months, *SD* = 8; 23 boys and 16 girls), who came from rehabilitation centers in Hebei and kindergartens in Beijing, participated in the current study. The hearing-impaired children had severe and congenital hearing loss. The characteristics of participants are shown in **Table 1**. There were no significant group differences in age (*t* = -0.889, *p* = 0.377) and gender (χ^2^ = 0.054, *p* = 0.817). The results of the PPVT-IV test (Dunn and Dunn, 2007) showed that the receptive vocabulary skill of hearing-impaired children was worse than that of hearing children (*t* = -18.26, *p* < 0.001). The hearing-impaired children received training of voice production and voice recognition for 1 h every day and they were encouraged to use oral language for daily communication. This study was reviewed and approved by the Ethics Committee of School of Psychology, Capital Normal University with the written informed consent signed by the parents of the participants prior to the experiment.

**Table 1:** Characteristics of participants in each group.

	Hearing-impaired	Hearing
Number of children	39	39
Mean age (SD) (months)	60 (12)	60 (8)
Range of age (months)	44–90	44–72
Mean age of using hearing aids (SD) (months)	29 (12)	//
Range of unaided-hearing loss (left/right)	60–125/60–125	//
Ratio of males: females	24:15	23:16

### Materials and Design

Twenty-four video clips were created. The actors were graduate students (*M*_age_ = 24.7 years) at Capital Normal University. Each clip began with a 4.5 s period in which the actor silently looked at the camera and exhibited either a neutral or a positive facial expression, followed by a 7.5 s period during which the actor delivered either a neutral statement (e.g., you are a child with hair) or a positive statement (e.g., you are a cute child) with a talking face and gazed at the camera (see Weeks et al., 2013 for similar video clips). For the videos, there were four conditions according to the congruency of visual vs. oral statements: 2 congruent (neutral expression/neutral statement, positive expression/positive statement) and 2 incongruent conditions (neutral expression/positive statement, positive expression/neutral statement). Each condition included six videos created by six actors (three males and three females). The faces of the different actors were normalized to a uniform size for presentation. The sounds of all statements in the videos were dubbed by the same person and were transferred (with cool edit Pro2.1 software) to male sounds or female sounds according to the actors’ gender. Sixty-five students were recruited to rate the emotional valence of the facial expressions and oral statements in the videos. The percentage of agreement was calculated relative to the emotional valence designated by experimenters. The results showed that the percentages of agreement for the emotional valence of the facial expressions and oral statements were above 95%.

### Apparatus and Procedure

Participants’ eye-movements were recorded by a TobiiX120 system at a rate of 120 Hz. Children were instructed to view 24 video clips presented randomly on a 21.5-inch Samsung monitor. The size of each video clip was 1280 × 720 pixels. The distance between the monitor and participants’ eyes was 75 cm. Before the experiment, all the participants completed the calibration with a nine-point grid. There was a central fixation cross for 2 s followed by a blank screen for 2 s before the presentation of each video clip. All children were orally told that “After looking at each central fixation cross, you will see a video with voice. Please watch and listen carefully.” To make sure that hearing-impaired participants could understand the instructions, their language teacher helped the experimenter to explain the instructions. The mean retention rate of the eye-movement data when viewing the video clips for hearing-impaired and hearing children were 92 and 96% respectively.

### Data Analysis

**FIGURE 1:**
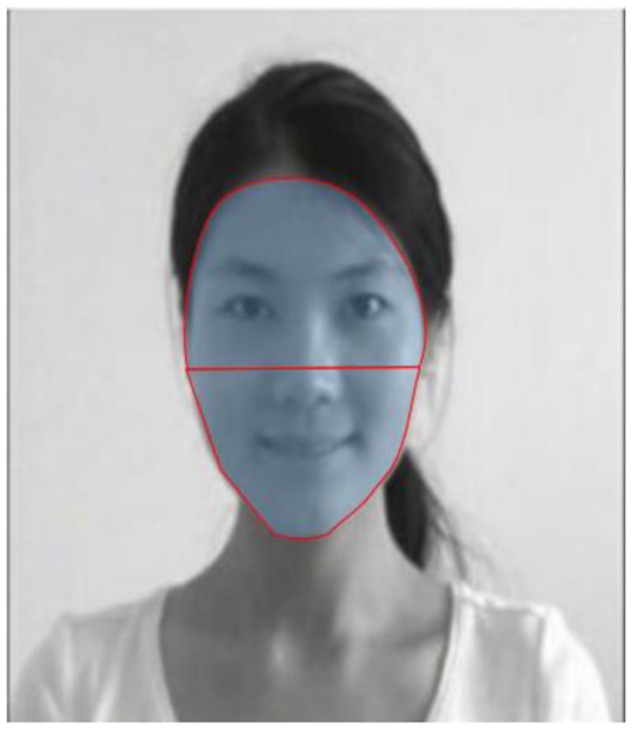
An example of AOIs.

We used an algorithm with dispersion threshold (maximum fixation radius = 1) and duration threshold (minimum fixation duration = 100 ms) criteria (Salvucci and Goldberg, 2000; Borgi et al., 2014) to determine fixations in the current study. The upper and lower parts of the face represented the generalized regions for eyes and mouth, respectively, and were designated areas of interest (AOIs) (see **Figure 1** for an example of AOIs). Trials (i.e., 6%) with no fixations on AOIs were not included in the analyses. The number of saccades between the upper and lower parts of the face, the number of fixations within the AOIs, and the viewing time (i.e., the sum of the individual fixation durations) within the AOIs were computed. The number of fixations and the viewing time were transformed to percentages by dividing the total number of fixations and the total viewing time on the stimuli, respectively. As the number of fixations and the viewing time in the upper and lower parts of the face were highly dependent on each other, we mainly report the patterns for the upper part of the face.

We analyzed the data in the period (4.5 s) with only the facial expression and the period (7.5 s) after the oral statement appeared. For each period, three repeated-measure ANOVAs (dependent variables: the number of saccades between the upper and lower parts of the face, the percentage for the number of fixations within the upper part of the face, and the percentage for the viewing time within the upper part of the face) were conducted with group as a between-subject factor, and the emotional valences of facial expression and oral statement were calculated as within-subject factors. All ANOVAs were calculated when age and gender were controlled.

## Results

### The Number of Saccades between the Upper and Lower Parts of the Face

**Table 2** illustrates the number of saccades between the upper and lower parts of the face for each condition. During the period with the facial expression only, no significant main effects or interactions were found (*ps* > 0.05).

**Table 2:** The mean (and standard deviations) for the number of saccades between the upper and lower parts of the face in each condition.

		NE-NS	NE-PS	PE-NS	PE-PS
Periods with facial expression	Hearing-impaired	1.7 (1.4)	1.7 (1.3)	1.8 (1.4)	1.6 (1.2)
	Hearing	1.9 (1.5)	1.9 (1.1)	2.2 (1.2)	2.1 (1.3)
Periods with oral statement	Hearing-impaired	1.9 (1.4)	1.9 (1.4)	1.8 (1.3)	1.6 (1.3)
	Hearing	2.5 (1.6)	2.5 (1.6)	2.6 (1.7)	2.1 (1.4)

*NE, neutral expression; NS, neutral statement; PE, positive expression; PS, positive statement.*

During the period with the oral statement, we observed a significant group effect, *F*(1,74) = 4.169, *p* = 0.045, η^2^ = 0.054, indicating that hearing children produced more inter-regional saccades as compared to hearing-impaired children. The other main effects and interactions during the period with the oral statement were not significant (*ps* > 0.05).

### The Percentage for the Number of Fixations within the Upper Part of the Face

**FIGURE 2:**
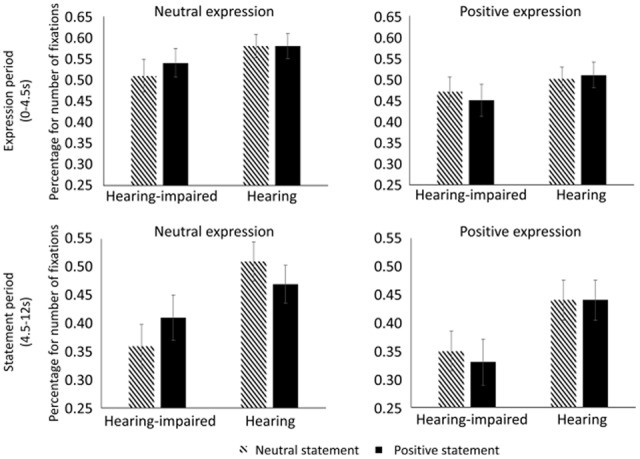
The percentage for number of fixations during the periods with facial expression **(top panel)** and oral statement **(bottom panel)** in each condition. The ordinate (Y axis) presents the quotient of the fixation number within the upper part of the face divided by the total fixation number on the stimuli.

**Figure 2** illustrates the percentage for the number of fixations within the upper part of the face for each condition. During the period with the facial expression only, no significant main effects or interactions were found (*ps* > 0.05).

During the period with the oral statement, we found that hearing-impaired children fixated less on the upper part of the face than hearing children, *F*(1,74) = 4.261, *p* = 0.043, η^2^ = 0.055. In addition, there was a significant three-way interaction among group, valence of expression, and valence of statement, *F*(1,74) = 5.055, *p* = 0.028, η^2^ = 0.065. Further analyses indicated that hearing-impaired children produced fewer fixations on the upper part of the face in the neutral expression/neutral statement condition relative to the neutral expression/positive statement condition (*p* = 0.033), whereas hearing children produced numerically more fixations in the neutral expression/neutral statement condition relative to the neutral expression/positive statement condition (*p* = 0.055). There was no significant difference between the positive expression/neutral statement condition and the positive expression/positive statement condition for either group (*ps* > 0.05). The other main effects and interactions during the period with the oral statement were not significant (*ps* > 0.05).

**FIGURE 3:**
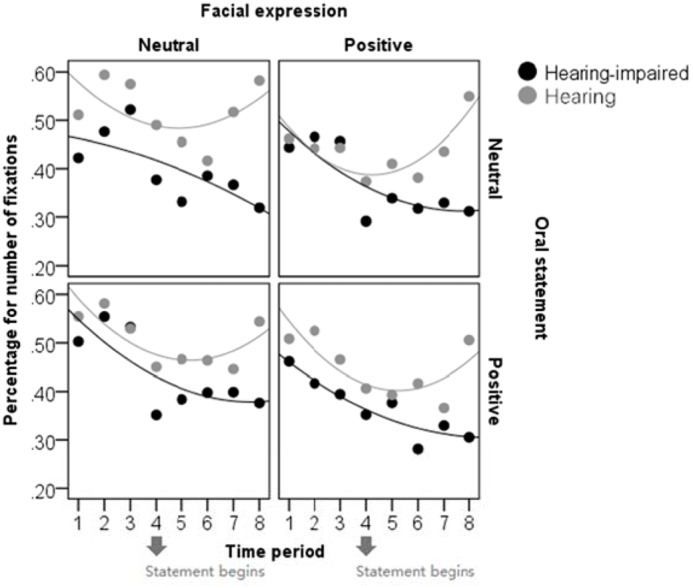
The scatter plots between time period and percentage for number of fixations in each condition. The abscissa (X axis) presents eight time periods with 1.5 s for each one. The ordinate (Y axis) presents the quotient of the fixation number within the upper part of the face divided by the total fixation number on the stimuli.

To illustrate the development of attention allocation as time went on during the trial, we divided the time into eight periods (1.5 s per period) and drew scatter plots between the time period and the percentage for number of fixations as a function of group, valence of facial expression, and valence of oral statement (see **Figure 3**). All the scatter plots were fitted by quadratic lines. Again, the patterns in the scatter plots showed that, after the oral statement was presented, hearing children looked more at the upper part of the face compared to hearing-impaired children and that hearing-impaired children looked less at the upper part of the face in the neutral expression/neutral statement condition relative to the neutral expression/positive statement condition.

### The Percentage for Viewing Time within the Upper Part of the Face

As shown in **Table 3**, the pattern of the percentage for viewing time was generally similar to the percentage for the number of fixations. During the period with the facial expression only, no significant main effects or interactions were found (*ps* > 0.05).

**Table 3:** The mean (and standard deviations) for the percentage of viewing time within the upper part of the face in each condition.

		NE-NS	NE-PS	PE-NS	PE-PS
Periods with facial expression	Hearing-impaired	0.50 (0.25)	0.55 (0.22)	0.47 (0.22)	0.44 (0.24)
	Hearing	0.61 (0.18)	0.61 (0.19)	0.51 (0.21)	0.53 (0.23)
Periods with oral statement	Hearing-impaired	0.33 (0.25)	0.37 (0.28)	0.32 (0.24)	0.30 (0.26)
	Hearing	0.49 (0.25)	0.46 (0.24)	0.41 (0.25)	0.45 (0.26)

*NE, neutral expression; NS, neutral statement; PE, positive expression; PS, positive statement.*

During the period with the oral statement, we found that hearing-impaired children spent a shorter amount of time on the upper part of the face than hearing children, *F*(1,74) = 4.652, *p* = 0.034, η^2^ = 0.060. In addition, there was a significant three-way interaction among group, valence of expression, and valence of statement, *F*(1,74) = 6.202, *p* = 0.015, η^2^ = 0.078. Further analyses indicated that hearing-impaired children produced shorter viewing times on the upper part of the face in the neutral expression/neutral statement condition relative to the neutral expression/positive statement condition (*p* = 0.085; a trend toward significant), whereas there was no significant difference in viewing time between the neutral expression/neutral statement and the neutral expression/positive statement conditions (*p* = 0.163) for hearing children. There was no significant difference between the positive expression/neutral statement condition and the positive expression/positive statement condition for either group (*ps* > 0.05). The other main effects and interactions during the period with the oral statement were not significant (*ps* > 0.05).

In addition, neither the age of participants nor the age of using hearing aids was correlated with eye-movement measures (the percentage for the number of fixations and the percentage for viewing time within the upper part of the face) (*p*s > 0.05), suggesting that the findings of gaze behaviors are not modulated by the age of participants and the age of using hearing aids.

## Discussion

The results showed that children with HAs have different gaze patterns in emotion perception relative to hearing children. Although there was no group effect or experimental effect on gaze patterns when there was only a facial expression, children with HAs produced fewer fixations and shorter viewing time on the upper part of the face and fewer inter-regional saccades than hearing children after the oral statement was presented. In addition, children with HAs produced fewer fixations and shorter viewing time on the upper part of the face for the neutral expression/neutral statement condition relative to the neutral expression/positive statement condition. These results indicated that children with HAs were less likely to explore different parts of the face and preferred to look at the lower part of the face when there was an oral statement. This result is consistent with previous studies on deaf people without assistive devices (Agrafiotis et al., 2003; Muir and Richardson, 2005; Letourneau and Mitchell, 2011). Although young children with HAs are encouraged and trained to use verbal-auditory communication, their gaze patterns during social interactions are still different from hearing controls.

According to the scatter plots, hearing children’s attention was also attracted to the lower part of the face by the presentation of the oral statement, but they looked back at the upper part of the face after that. These typical gaze patterns suggest that eye contact plays an important role in emotion perception and social interaction. The behavior of keeping eye contact is inborn (Farroni et al., 2002). Gaze behavior and eye contact are a conspicuous aspect of human interaction and the eye region is often used as a cue for the attribution of emotional/mental states to others (Kleinke, 1986), an ability referred to as “theory of mind” (Premack and Woodruff, 1978). Early social experience, in turn, affects the development of eye gaze processing (Corkum and Moore, 1998; Senju and Csibra, 2008; Senju et al., 2015). Children with HAs have experienced auditory deprivation during early childhood, so they may not receive enough social interactions depending on oral communications that are necessary to understand the emotional expressions and situations (Rieffe and Terwogt, 2000). To compensate for deficits in oral communications, children with HAs may use a different gaze strategy to integrate visual cues which are helpful in understanding the minds of others. For instance, hearing-impaired children might look at the lower face in order to attempt to lip read, which is the skill of processing speech from the visible movements of the mouth (Campbell et al., 1986). Lipreading has the potential to be useful in phonological processing when there is a lack of hearing (Kyle et al., 2016). It is also possible for hearing-impaired people to incorporate upper face information through peripheral vision, as previous studies have indicated that deaf people have better peripheral vision (Codina et al., 2017). Although children with HAs may notice the upper face information according to peripheral vision, the peripheral processing is not efficient for detecting the details of the eyes. On these grounds, the adapted gaze patterns, which are caused by language delay and deficits in oral communications for children with HAs, can result in reduced opportunities to process the detail information of the eyes. As eye contact is very important for the attribution of emotional states to others, the altered eye gaze pattern may further lead to deficits in emotion perception delay.

Interestingly, children with HAs increased their attention on the upper part of the face when there was a positive oral statement after a neutral facial expression, compared to when there was a neutral oral statement after a neutral facial expression. The different gaze patterns between visual-auditory congruent conditions and incongruent conditions indicate that children with HAs can notice both auditory and visual information in understanding emotional expression. When the facial and voice cues were incongruent, children with HAs directed their eyes to the upper part of the face for confirmation. There has been debate on whether people with HAs or CIs can make adequate use of auditory information in response to incongruent visual-auditory stimuli (Schorr and Knudsen, 2005; Zupan and Sussman, 2009). Most et al. (1993) and Most and Aviner (2009) demonstrated that hearing-impaired adolescents with HAs or CIs relied on visual information but not auditory information in the perception of emotional expressions. With a different paradigm and younger participants, the present study found that both auditory and visual information could be used by hearing-impaired children with HAs to interpret emotional expressions. However, children with HAs may not skillful in matching the auditory and visual information, which results the inconsistency of gaze behavior between them and hearing children. When there was a neutral oral statement after a neutral facial expression, children with HAs would not move theirs gazes back to others eyes for further confirm. The lack of eye contact may give rise to a potential risk in development of emotion perception.

Much of the literature on children with CIs or HAs has focused on the effectiveness of these devices on auditory development and the perception of speech (Sharma et al., 2002; Lee et al., 2010). Now some researchers have begun to explore the broader effects of the use of CIs or HAs on children’s emotional and social development. Actually, these two research fields are not independent of each other. For instance, the promotion of speech for children with CIs or HAs seems to improve language-based concepts related to emotion (Rieffe and Terwogt, 2000; Dyck and Denver, 2003; Peters et al., 2009). The present study further demonstrated that the gaze patterns on facial expressions for children with HAs deviated from that for hearing children when additional cues were presented in the form of speech. These results indicate that the speech perception and emotion perception of children with HAs influence each other interactively. The absence of reliable effects during the period with only a static facial expression implies that the deviation of the gaze pattern for hearing-impaired people is different from that for autism spectrum disorder and social anxiety disorder. That is, the gaze avoidance of hearing-impaired people may be attributable to more complex reasons that are related to speech perception. Notably, as we used limited types of facial expressions (neutral and positive) and limited periods with only static facial expressions, whether hearing-impaired people have different gaze patterns for static facial expressions remains open to further investigation.

Much of the research on the recognition of emotional facial expressions has been conducted in ways that minimize context information, but emerging literatures have shown that context is encoded and required during emotion perception (e.g., Barrett and Kensinger, 2010). A completely context-free presentation of facial expressions is impossible in daily life and face muscle movements are insufficient for perceiving internal emotion. At around 3 years, children are able to express their emotions orally and understand situations and circumstances about emotions (Denham et al., 1994; Brown and Dunn, 1996). Although the present study has emphasized the linguistic context in the perception of emotional facial expressions, observers can attribute emotions more accurately according to non-verbal social context such as postures, gestures, and a-priory knowledge about the situation and the protagonist (Wiefferink et al., 2013; Hess and Hareli, 2015). Non-verbal social context might be particularly useful in emotion perception for hearing-impaired individuals. As the integration of facial expression and various context information would help young children to interpret other’s emotion, the role of the context in emotion perception need to be further investigated.

Due to the absence of auditory signal, the cortical reorganization of auditory-visual systems for hearing-impaired individuals has been frequently observed in previous studies without considering emotional aspects (e.g., Doucet et al., 2006; Campbell and Sharma, 2016). Besides the auditory-visual systems, more extensive brain regions are involved in face-voice emotional matching. For instance, the posterior superior temporal sulcus is considered as the neural basis for gaze, facial expression, and lipreading (Haxby et al., 2000; Nummenmaa et al., 2009) and a “multisensory” region for face-voice integration (Watson et al., 2014). Limbic structures, such as the amygdala, is associated with emotional processing (Dolan et al., 2001; Klasen et al., 2011). In the present study, the altered gaze patterns in auditory-visual perception of emotion for hearing-impaired individuals suggest that the cortical reorganization might also happen among sensory-, gaze-, and emotion-related brain regions. Investigation on the interactive influences among these regions will provide an insight into the underlying cortical mechanism of the reduced eye gaze for hearing-impaired children.

## Conclusion

The present study revealed the special gaze patterns in auditory-visual perception of emotion for children with assistive devices. While hearing children paid more attention to the upper part of the face, children with HAs paid more attention to the lower part of the face after the speech of emotional expression was presented, especially for the neutral facial expression – neutral oral statement condition. The negative cause and effect of overlooking the upper part of the face, especially in the neural expression/neural statement condition, should be avoided in earlier rehabilitation for hearing-impaired children with assistive devices.

## Ethics Statement

All procedures performed in the study involving human participants were conducted in accordance with the ethical standards of the institutional and national research committee and with the 1964 Helsinki declaration and its later amendments or comparable ethical standards. Written informed consent was obtained from all participants included in the study.

## Author Contributions

YW: substantial contributions to the conception or design of the work. WZ: drafting the work or revising it critically for important intellectual content and final approval of the version to be published. YC: acquisition, analysis of data for the work. XB: analysis and interpretation of data for the work.

## Conflict of Interest Statement

The authors declare that the research was conducted in the absence of any commercial or financial relationships that could be construed as a potential conflict of interest.
